# A Meta-Analysis of the Influence on Inflammatory Factors in Type 2 Diabetes among Middle-Aged and Elderly Patients by Various Exercise Modalities

**DOI:** 10.3390/ijerph20031783

**Published:** 2023-01-18

**Authors:** Weijun Yang, Haotian Jiao, Yizhang Xue, Lishuo Wang, Ye Zhang, Boqian Wang, Ziyi Teng, Junyan Li, Haotian Zhao, Chang Liu

**Affiliations:** 1 Sports Coaching College, Beijing Sport University, Beijing 100084, China; 2 School of Education, Beijing Sport University, Beijing 100084, China; 3 School of Management Engineering and E-Commerce, Zhejiang Gongshang University, Hangzhou 310018, China; 4 Tourism Management, Beijing Sport University, Beijing 100084, China; 5 School of Sport Science, Beijing Sport University, Beijing 100084, China; 6 Department of Physical Education, Jiangnan University, Wuxi 214122, China

**Keywords:** type 2 diabetes, training modalities, inflammation, meta-analysis, middle-aged and elderly patients

## Abstract

The purpose of this study was to investigate the effects of various exercise modalities on inflammatory factors in middle-aged and elderly patients with type 2 diabetes (MEPT2D), as lifestyle changes, such as physical activity and dietary modifications, are considered important in the prevention of type 2 diabetes. For the study methodology, Pubmed, CNKI, EBSCO, Wanfang Data, and Web of Science were selected for the search. The methodological quality of the included studies was assessed by the Cochrane Risk of Bias (ROB) tool, and statistically analyzed using the RevMan 5.4.1 analysis software, which included 18 investigations involving 853 study subjects. Meta-analysis findings indicated that aerobic training (AT), resistance training (RT), combined training (CT), and high-intensity interval training (HIIT) showed significant reductions in CRP, TNF-α, IL-6, and IL-10 levels in MEPT2D. Among them, HIIT was superior to other training modalities in reducing TNF-α levels, while CT was superior to AT, RT, and HIIT in decreasing IL-6, IL-10, and CRP in MEPT2D. Meanwhile, RT had limited effects in reducing CRP and TNF-α levels in MEPT2D. However, HIIT had no significant effect on IL-6 and IL-10 in MEPT2D. In conclusion, long-term regular AT, RT, CT, and HIIT all contributed to the reduction of inflammatory status (CRP, TNF-α, IL-6, and IL-10) in MEPT2D, while CT (for CRP, IL-6, and IL-10) and HIIT (for TNF-α) represent the best approaches to counteract the inflammatory response in MEPT2D.

## 1. Introduction

Diabetes is an epidemic and pathologically complex chronic disease problem in the world today that affects patients of almost all ages. According to the World Diabetes Federation (IDF) and the World Health Organization (WHO), the number of adults with diabetes reached 537 million worldwide in 2021. This represents an increase of 74 million (or 16%) compared with 2019, highlighting the alarming growth in the global prevalence of diabetes, which the IDF predicts will reach 783 million by 2045, an increase of 46%, more than twice the projected population growth (20%) over the same period, with one in eight adults worldwide likely to have diabetes [1]. Diabetes is associated with many major cardiovascular diseases, including heart failure, coronary artery disease, ischemic heart disease, peripheral artery disease, and stroke, all of which increase with the age of the patient [2]. For type 2 diabetes, which is characterized by insulin resistance and is the precursor type of diabetes (more than 90%), a large number of studies and clinical practices have confirmed that it is closely associated with risk factors for chronic kidney disease, eye disease, cardiovascular disease, and other disorders. Patients with type 2 diabetes have a higher cardiovascular risk than non-diabetic patients, especially among middle-aged and even older patients with type 2 diabetes, whose risk is always two to three times higher [3]. Type 2 diabetes increases atherosclerosis, coronary artery disease, cerebral atherosclerosis, stroke, kidney failure, retinopathy, cataracts, diabetic foot, menstrual disorders, hypertension, and metabolic syndrome in patients. In addition, patients with diabetes may develop collateral psychological problems such as depression and anxiety disorders [4]. To date, interventions to prevent type 2 diabetes have focused on changing the behavioral structure of life, such as increasing physical training or restructuring daily diets.

Persistent abnormal blood glucose levels linked to type 2 diabetes may contribute to a long-term systemic inflammation response [5,6]. This further worsens the patient’s pathological environment, trapping the cardiovascular, cerebrovascular, and neurological health of type 2 diabetics in a vicious cycle. Currently, a significant proportion of people with type 2 diabetes worldwide fail to fulfill the recommended exercise prescription of one hour of moderate intensity physical activity per day [7]. However, training was effective in improving blood glucose levels and ROS concentrations as well as suppressing the systemic inflammatory response [8]. Although training at different intensities and modalities has been demonstrated to be efficacious in diminishing TNF-a and inflammatory cellular factors such as CRP and IL-6 and IL-10, there is some variability among the current studies regarding this outcome [9].

Although differences in antioxidant levels in skeletal myocytes across subjects of different ages may be the main source of the varying outcomes of these studies, differences in the subjects’ improved anti-inflammatory capacity due to different levels of upregulation in glutathione peroxidase, catalase, and superoxide dismutase [10]. More specifically, being an acute-phase protein, CRP is primarily synthesized by hepatocytes and is triggered in the presence of altered levels of pro-inflammatory cytokines induced during the period of infection/inflammation. For this reason, it is considered a reliable biological indicator of the non-specific acute inflammatory phase, however, some prospective investigations have revealed that it is also involved in a range of chronic inflammatory responses, such as Parkinson’s disease, age-related macular degeneration, Alzheimer’s disease, hemorrhagic stroke, and cardiovascular disease [11].

Recent findings, however, suggest a more important and critical function of tumor necrosis factor-alpha (TNF-α) as a pathophysiological component of autoimmune diseases, in addition to its capacity to induce tumor necrosis. Through this pathway, a series of crucial cellular responses are initiated, covering cell proliferation, survival, and differentiation. Nevertheless, an aberrant activation state of the TNF-α signaling pathway may lead to or exacerbate chronic inflammation in vivo, which may subsequently evolve into a variety of pathological complications, such as automatic immune diseases, and in patients with type 2 diabetics, hyperactive TNF-alpha can cause even more severe insulin resistance, which may worsen the disease [12]. IL-10 and IL-6 are both members of the cytokine family that promote inflammation and induce a variety of related pathological responses. IL-10 is a multifunctional chemokine that exerts immune-suppressive and immune-stimulatory effects in a wide spectrum of cell types, thereby regulating inflammatory and immune responses. It has critical functions in the tumor environment, immune regulation of the lymphatic and hematopoietic systems, and is closely associated with diseases of the blood, digestive system, and especially the cardiovascular system. IL-6 is implicated in the differentiation and growth regulation of most cells, and it plays an influential role in the acute phase of the body’s anti-infective immune response, regulatory immune responses, and hematopoiesis, but also has a pathological role in chronic inflammation and in the persistent dysregulation of IL-10 and IL-6 synergistic responses in the autoimmune system [13].

The main different training modalities of interest in this study include aerobic training (AT), resistance training (RT), aerobic combined with resistance training (CT), and high intensity interval training (HIIT). AT is a form of physical activity that relies on aerobic metabolism to provide the energy required for the exercise process, and a form of exercise that relies primarily on the provision of oxidative energy to generate energy and support the activity process, accomplished through the rhythmic repetition of a series of physical activities ranging from mild to moderate intensity over a certain period of time, such as jogging, cycling, swimming, aerobics, etc. The intensity of AT is usually linearly related to the maximum oxygen uptake (VO_2_max). RT is an active movement of the muscles to overcome external resistance and is a physical training activity whose main objective is to restore and develop muscle mass. It includes various training methods such as isotonic and isometric training. The training movements are usually related to weightlifting and are a mode of weightlifting performed while keeping the speed of the movement essentially constant, for example: squat, push-up, dumbbell fly, bench press, barbell rowing, and other movement. The intensity of resistance training is generally related to the weight, trajectory, number of movements, and time of a single session. CT is a training modality based on aerobic and anaerobic (resistance) cross-training, with a structured training program that fully mobilizes the trainer’s aerobic and anaerobic energy supply systems and basal metabolic levels. As an integrated training modality, the training content is more flexible and the movements usually consist of a series of compound movements with aerobic movements as a warm-up and aerobic movements interspersed with intervals of anaerobic movements. The intensity of the training is usually assessed by the VO_2_max of the aerobic exercise and the total training load of the anaerobic training. High Intensity Interval Training (HIIT) is a complex training modality where high intensity, multi-form anaerobic, and aerobic training is alternated with short bursts of energy recovery until exhaustion to maximize the use of the anaerobic energy release system [14], which mainly involves the rapid cycling of a series of high intensity movements involving multiple major muscle groups throughout the body in one set, such as open jump, push-up with alternating knee tuck, and a bow and arrow squat. The purpose of this study was to compare the effects of inflammatory factors in middle-aged and elderly patients with type 2 diabetes (MEPT2D) in a cross-sectional manner, and to provide a credible theoretical reference for selecting a reasonable training modality in practice to better improve the systemic chronic inflammatory response-related diseases in MEPT2D.

## 2. Materials and Methods

### 2.1. Search Strategy

The study rigorously followed the Preferred Reporting Items for Systematic Evaluation and Meta-Analysis (PRISMA) guidelines for the literature search. The keywords “middle-aged and elderly patients with type 2 diabetes”, “exercise intervention”, “aerobic training”, “resistance training”, “aerobic combined with resistance training”, “high-intensity interval training”, “inflammatory factor”, “inflammation”, and “inflammatory response” were used to obtain the literature by searching PubMed, Wanfang database, Web of Science, China National Knowledge Structure, and EBSCO, while the language of the literature was limited to English. The search strategy used a combination of subject terms and free words, and also systematically tracked references related to the cited literature, whereas the search dates ranged from January 2000 to October 2022.

### 2.2. Criteria for Inclusion and Exclusion of the Literature

Inclusion criteria: (1) Sample selection criteria: middle-aged (40–55 years old) and elderly (above 55 years old) type 2 diabetic patients (above 40 years old) with BMI ≤ 42 and disease duration ≥ 1 year; (2) Type of study: randomized controlled trial or self-control trial; (3) Subjects: participants were type 2 diabetic patients without any cardiovascular or metabolic disease and physical impairment; (4) Results: outcome indicators consisted of IL-6, IL-10, TNF-α, and CRP.

Exclusion criteria: (1) type 2 diabetes or other types of diabetes in different age groups; (2) publications lacking complete articles or with ambiguous experimental data; (3) grey literature, such as conference abstracts, papers, case studies, and reviews; and (4) duplicate publications.

### 2.3. Study Selection and Data Extraction

Citations management software from Endnote 20.2.1 for facilitating the filtering of literature data. Extracts of outcome evaluation indicators included the first author and publication date of the literature, sample size, age, gender, training format, intervention method (training modality, periodicity, duration, frequency, with or without pharmacological intervention, etc.). The literature screening and data extraction were accomplished independently by two investigators (WJY, HTJ) and cross-checked upon completion. In case of disagreements emerged during the discussion, a third investigator (CL) was invited for more detailed deliberation and decision.

### 2.4. Quality Evaluation

Literature quality was measured using the Cochrane Risk of Literature Bias tool in Review Manager 5.4.1 (java 8 64-bit) analysis software across six metrics: selective bias, measurement bias, implementation bias, attrition bias, and other biases. The evaluation outcomes include low risk (high quality), uncertainty, and high risk (low quality). Studies were defined as low risk and high quality if five indicators were low risk, medium quality if two or a greater number of indicators were inconclusive, and high risk if two or a great number of indicators were high risk. The quality assessment was done by two investigators (WJY, HTJ) independently, and we would transfer to a third investigator (CL) for further consideration and decision if there was no consensus reached on the results.

### 2.5. Analytical Statistics

Review Manager 5.4.1 was used to analyze the data. The effect sizes of the studies were expressed as WMD (weighted mean difference) and 95% confidence intervals were calculated. The heterogeneity of the study was measured by the consistency coefficients *p* and i. In other words, if the results show *p* > 0.10 and i < 50%, the group of studies is indicated to be homogeneous and is analyzed using a fixed effects model; if not, a random effects model is applied. A comparison of different training modalities was also performed by subgroup analysis. The effect of bias on the overall effect of individual studies with differences were eliminated by sensitivity analysis. If there was statistical heterogeneity between study groups rather than clinical heterogeneity, a random-effects model was used for analysis. Descriptive analysis was used to address situations where study heterogeneity was too pronounced to determine the cause. Publication bias was defined by using funnel plots where the number of studies exceeded 10, a = 0.05 was selected as the significance test level for the study.

## 3. Results

### 3.1. General Findings of Selected Research Literature

**Figure 1:**
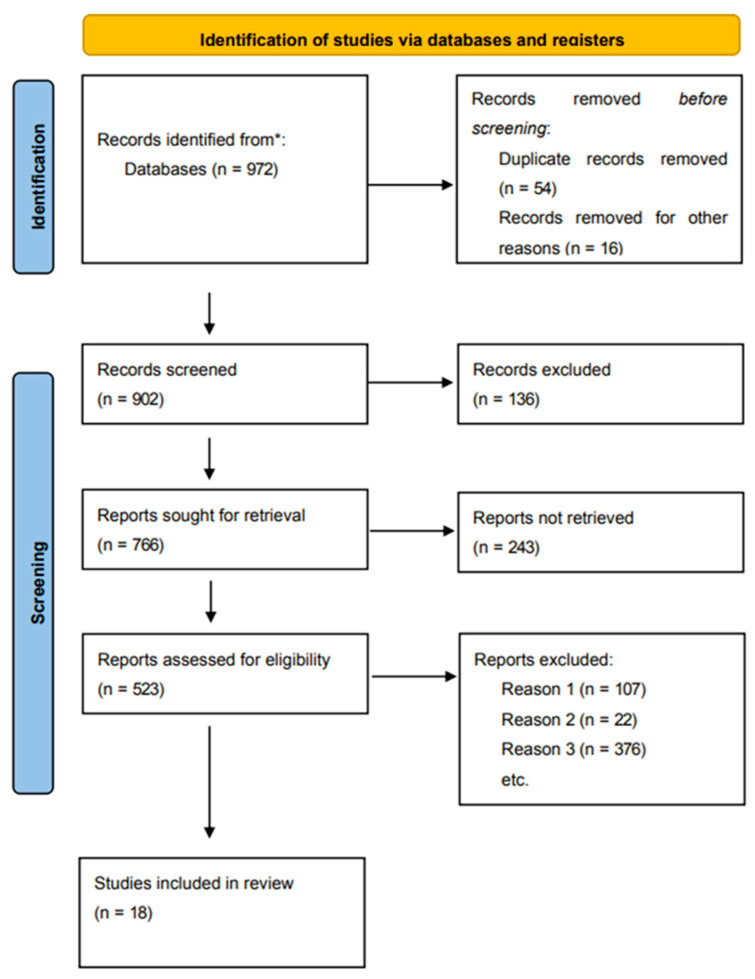
Flow chart of literature screening. *: Selected data sources Pubmed, CNKI, EBSCO, Wanfang Data, and Web of Science.

Figure 1 illustrates the detailed literature exclusion and selection procedure. Ultimately, eighteen experimental studies were incorporated into the search and screening of 972 publications.

### 3.2. The General Characteristics of Selected Research Literature

Eighteen randomized controlled trials were selected, including 853 subjects. Nine of the studies contained the AT group, six studies included the RT group, nine studies included the CT group, and only three studies contained the HIIT group. By country, Brazil, Italy, Australia, and Saudi Arabia each had three studies, while Iran, Greece, China, Korea, Japan, and Portugal each had one study. During the experimental design, different amounts of MEPT2D were recruited through clinical screening, internet recruitment, and leaflet solicitation, and randomly assigned to experimental or control groups on the basis of basic equality, and were observed and trained in the experimental or control groups. The experimental group engaged in a training program with a different experiential design component; in contrast, participants in the control group kept their normal rhythm of life but without the intervention of exercise. Subjects were then measured for various inflammatory response markers, including CRP, TNF-α, IL-6, and IL-10. Participants remained largely homogeneous in terms of gender and age, i.e., samples from the experimental and control groups were largely balanced in terms of gender and similar in age, with the specific characteristics shown in Table 1.

### 3.3. Quality Assessment of the Selected Literature

**Figure 2:**
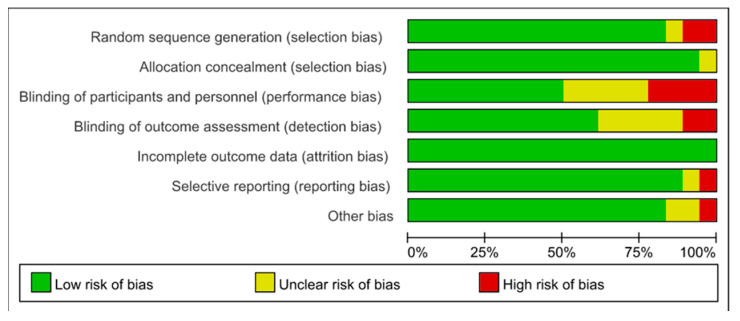
The bias of the included studies.

**Figure 3:**
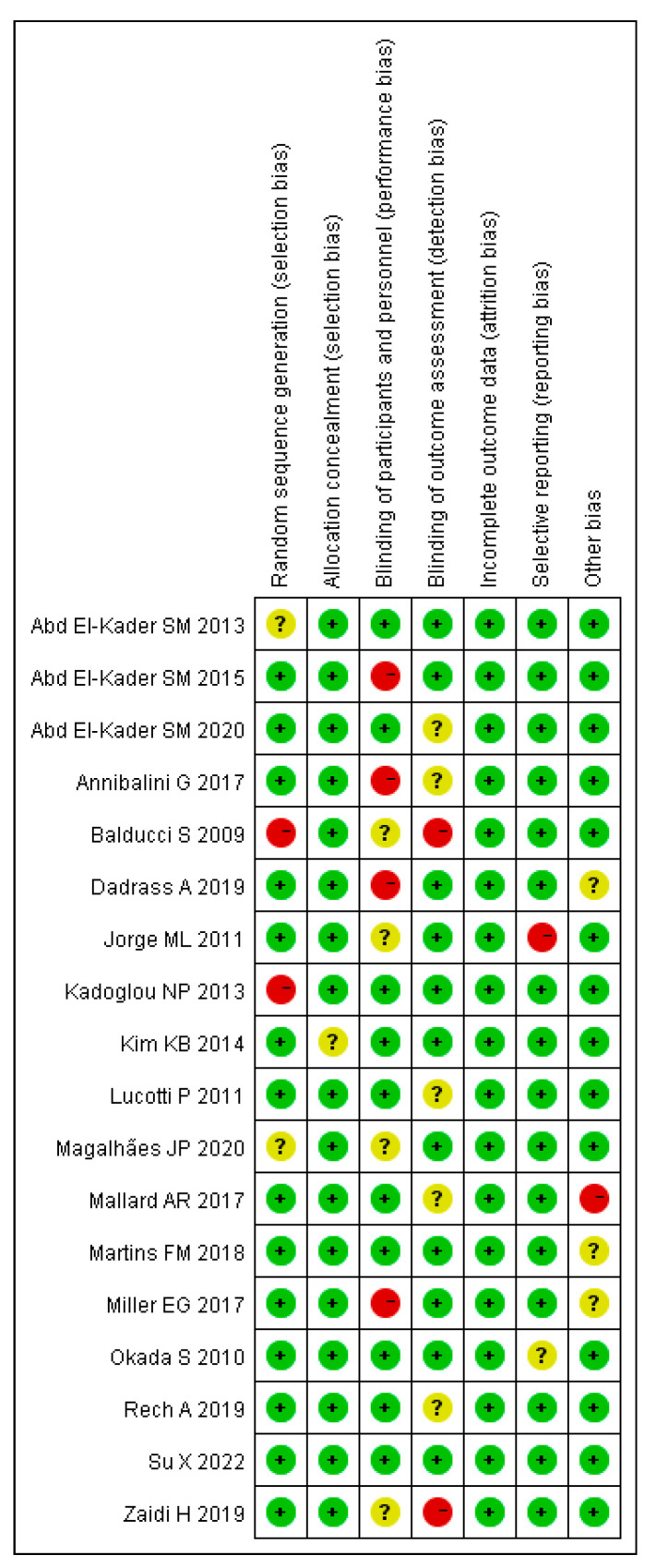
Risk of bias summary.

The quality of the literature and the risk of investigation bias were measured by Cochrane’s Risk of Bias tool for 18 selected studies, which comprised one high-risk study, two medium-risk studies, and fifteen low-risk studies, as presented in Figure 2 and Figure 3.

### 3.4. Meta-Analysis of the Effects of Various Exercise Modalities on CRP Levels in MEPT2D

**Figure 4:**
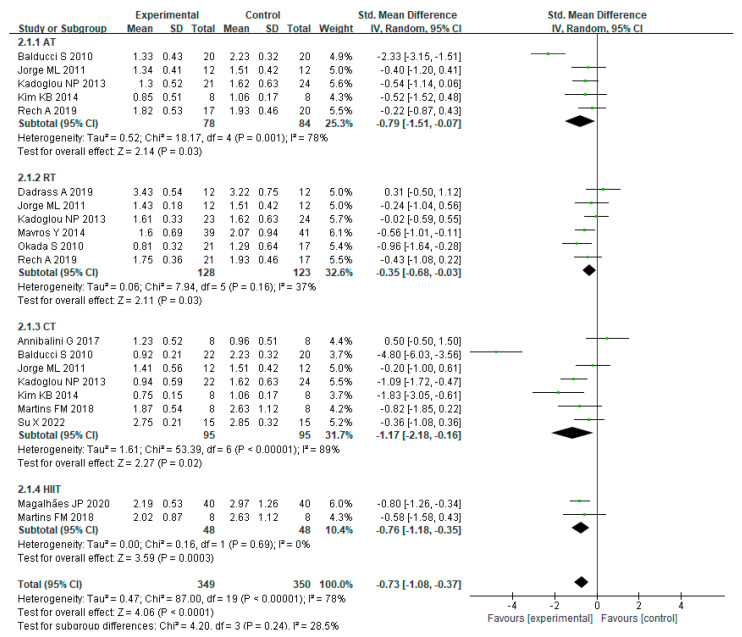
The effects of various exercise modalities on CRP levels in MEPT2D.

For the use of a random effects model to study the effect of various exercise modalities on CRP levels in MEPT2D, heterogeneity existed among the studies (I² = 78%, *p* < 0.00001). The combined effect scale SMD = −0.73, 95% CI = −1.08 to −0.37 (*p* < 0.0001), showed that various training modalities (AT, RT, CT, and HIIT) were effective in decreasing CRP levels in MEPT2D. Subgroup analysis showed that AT (SMD = −0.79, 95% CI = −1.51 to −0.07, *p* = 0.03), CT (SMD = −1.17, 95% CI = −2.18 to −0.16, *p* = 0.02) and HIIT (SMD= −0.76, 95% CI = −1.18 to −0.35, *p* = 0.0003) were superior to RT (SMD = −0.35, 95% CI = −0.68 to −0.03, *p* = 0.03) in reducing CRP levels, whilst CT was the most effective in reducing CRP levels (Figure 4).

### 3.5. Meta-Analysis of the Effects of Various Exercise Modalities on TNF-α Levels in MEPT2D

**Figure 5:**
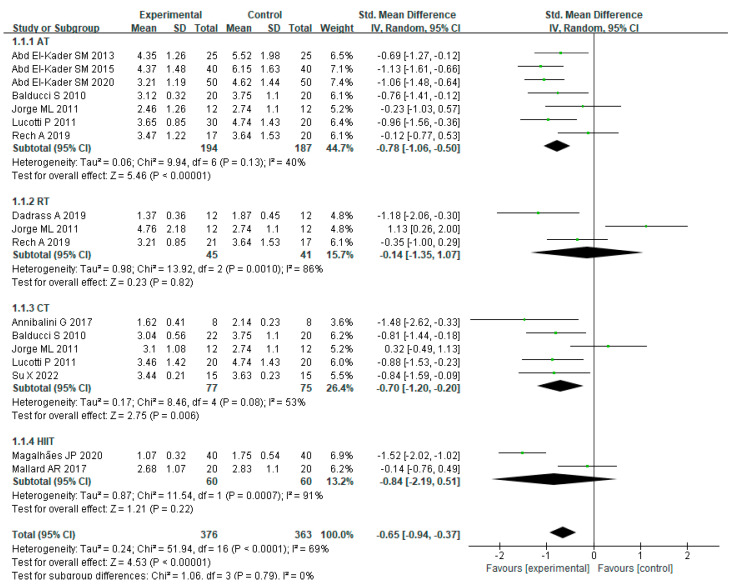
The effects of various exercise modalities on TNF-α levels in MEPT2D.

For the effect of various exercise modalities on TNF-α levels in MEPT2D, there was heterogeneity amongst these studies (I² = 69%, *p* < 0.0001). Using a random effects model, the combined effect scale SMD =−0.65, 95% CI =−0.94 to −0.37 (*p* < 0.00001), indicated that exercise training reduced serum TNF-α levels in MEPT2D. The subgroup analysis showed that AT (SMD = −0.78, 95% CI = −1.06 to −0.50, *p* < 0.00001), RT (SMD = −0.14, 95% CI = −1.35 to 1.07, *p* = 0.82), CT (SMD = −0.70, 95% CI = −1.20 to −0.20, *p* = 0.006), and HIIT (SMD = −0. 84, 95% CI = −2.19 to 0.51 *p* = 0.22) all reduced TNF-α levels in MEPT2D, but RT (SMD = −0.14, 95% CI = −1.35 to 1.07, *p* = 0.82) had a limited effect on decreasing TNF-α. Among them, HIIT was the most effective in reducing TNF-α (Figure 5).

### 3.6. Meta-Analysis of the Effects of Various Exercise Modalities on IL-6 Levels in MEPT2D

**Figure 6:**
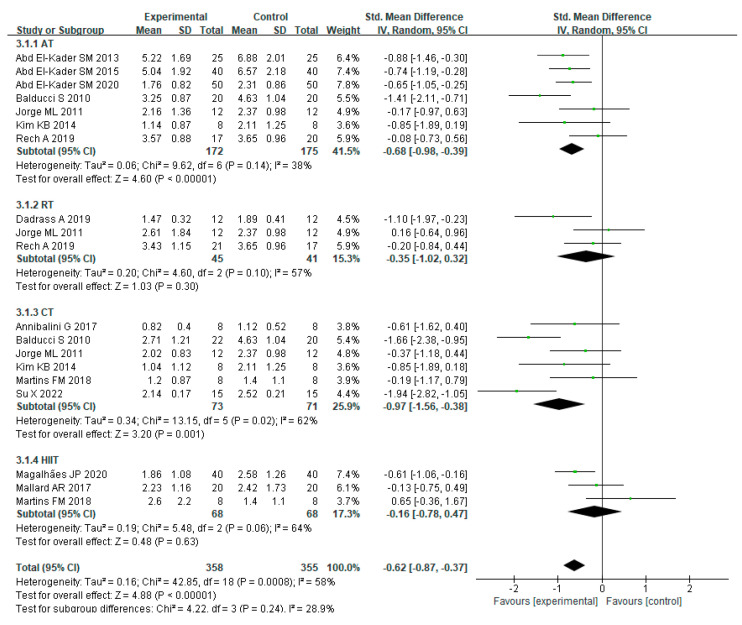
The effects of various exercise modalities on IL-6 levels in MEPT2D.

In various studies, there had been heterogeneity in the effects of varying training approaches on IL-6 levels in MEPT2D (I² = 58%, *p* = 0.0008). Using a random effects model, the combined effect scale SMD = −0.62, 95% CI = −0.87 to 0.47 (*p* < 0.00001), indicating that multiple forms of exercise reduce IL-6 levels in MEPT2D. Subgroup analysis showed that the AT group (SMD = −0.68, 95% CI = −0.98 to 0.39, *p* < 0.00001), RT group (SMD = −0.35, 95% CI = −1.02 to 0.32, *p* = 0.30), CT group (SMD = −0.97, 95% CI = −1.56 to −0.38, *p* = 0.001), and HIIT group (SMD = −0.16, 95% CI = −0.78~0.47, *p* = 0.63) all reduced IL−6 levels, but HIIT had a relatively limited effect on IL-6 levels, while the CT group had the best effect on IL-6 levels among the multiple training modalities (Figure 6).

### 3.7. Meta-Analysis of the Influence on IL-10 Levels in Type 2 Diabetes among Middle-Aged and Older Patients by Various Exercise Modalities

**Figure 7:**
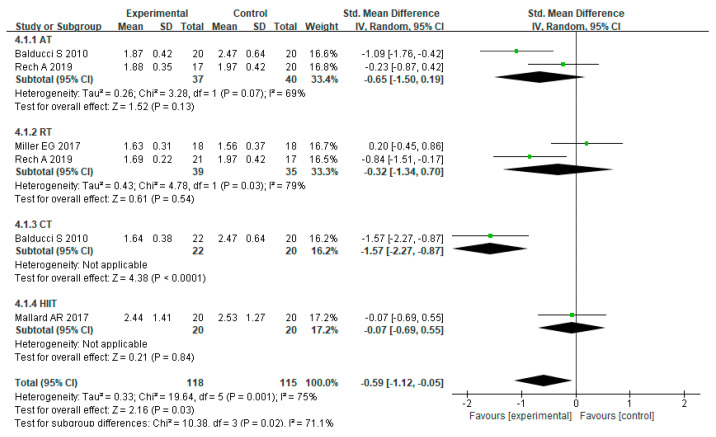
The effects of various exercise modalities on IL-10 levels in MEPT2D.

For the effect of different training modalities on IL-10 levels in MEPT2D, heterogeneity was observed across studies (I² = 75%, *p* = 0.001). Using a random effects model, the combined effect scale SMD = −0.59, 95% CI = −1.12 to −0.05 (*p* = 0.03), indicated that exercise training could reduce IL-10 levels to some extent in MEPT2D. Subgroup analysis showed that AT group (SMD= −0.65, 95% CI= −1.50 to 0.19, *p* = 0.13), RT group (SMD = −0.32, 95% CI = −1.34 to 0.70, *p* = 0.54), and CT group (SMD = −1.57, 95% CI = −2.27 to −0.87, *p* < 0.0001) both reduced IL-10 levels, and HIIT had a limited effect on IL-10 (SMD = −0.07, 95% CI = −0.69 to 0.55, *p* = 0.84) levels, while the CT group had the best effect on decreasing IL-10 levels (Figure 7).

### 3.8. Publication Bias and Sensitivity Analysis

**Figure 8:**
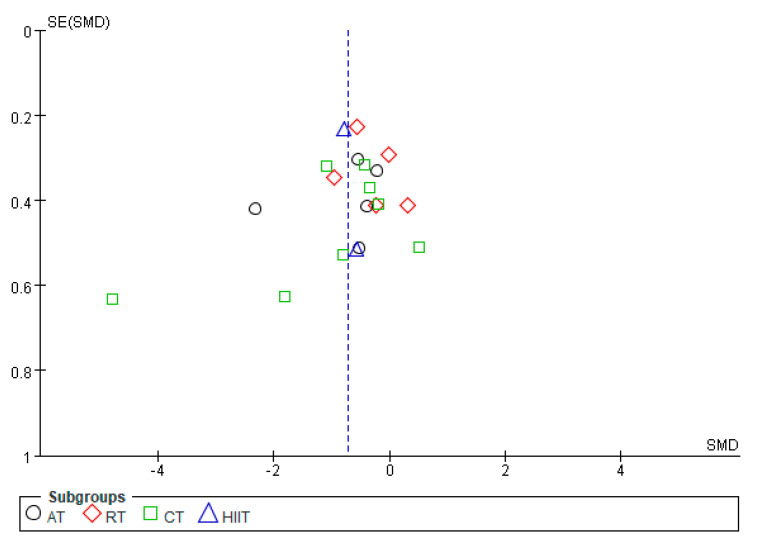
Publication bias of the effects of various exercise modalities on CRP levels in MEPT2D.

**Figure 9:**
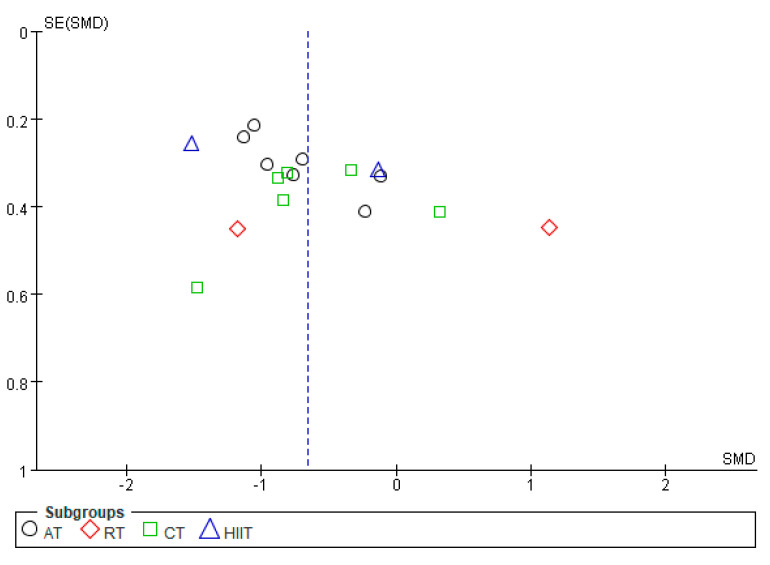
Publication bias of the effects of various exercise modalities on TNF-α levels in MEPT2D.

**Figure 10:**
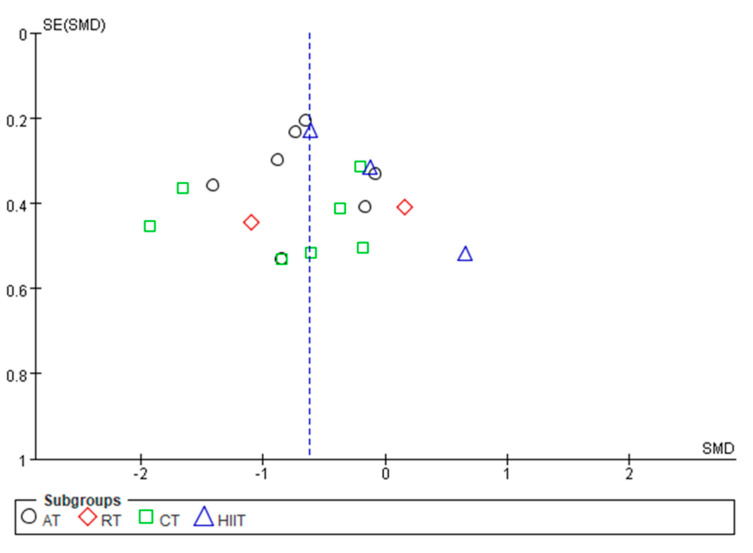
Publication bias of the effects of various exercise modalities on IL-6 levels in MEPT2D.

**Figure 11:**
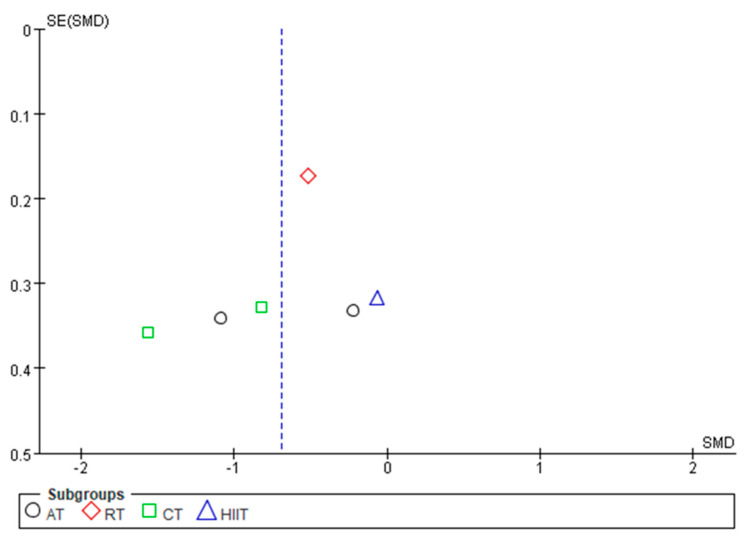
Publication bias of the effects of various exercise modalities on IL-10 levels in MEPT2D.

By mapping the funnel plots of CRP, TNF-α, IL-6, and IL-10 indicators, the distribution of the funnel plots of CRP, TNF-α, and IL-6 were found to be basically symmetrical, whereas that of IL-10 was basically asymmetric (Figure 8, Figure 9, Figure 10 and Figure 11). Analysis of sensitivity was performed by altering the joint effects scales. For the metrics mentioned above, studies were eliminated in isolation prior to re-running the meta-analysis. Compared with the results of previous studies, no significant changes were found, which suggests that the results of meta-analysis are consistent and the findings of this study are robust.

## 4. Discussion

In this study, 18 studies with 853 subjects were encompassed. The meta-analysis of the inflammatory factor data from subjects showed that exercise training considerably lowered IL-6, IL-10, CRP, and TNF-α levels in MEPT2D. The subgroup analysis of various modalities of training indicated that HIIT was superior to other training modalities in reducing TNF-α levels, while CT was superior to AT, RT, and HIIT in decreasing IL-6, IL-10, and CRP in MEPT2D, which is in agreement with the study of N.P.E. Kadoglou et al. [20]. Specifically, RT had only a limited effect on reducing CRP and TNF-α levels in MEPT2D. However, HIIT had no significant effect on IL-6 and IL-10 in MEPT2D. In conclusion, long-term regular AT, RT, CT, and HIIT all contributed to the reduction of inflammatory status (CRP, TNF-α, IL-6, and IL-10) in MEPT2D, while CT (for CRP, IL-6, and IL-10) and HIIT (for TNF-α) represent the best approaches to counteract the inflammatory response in MEPT2D.

This may be due to the fact that CT is superior to AT, RT, and HIIT in regulating glucose and lipid metabolism and improving the energy supply system, which produces more metabolic waste. In addition, CT has been shown to have a greater impact on physiological composition ratios than individual training modalities. In return, a more standard physiological composition might further alleviate the state of permanent inflammation due to the imbalanced glucose levels and high body fat percentage in MEPT2D [24]. The high heterogeneity of CRP, TNF-α, IL-6, and IL-10 in this study, as well as the variability of actions, patterns, and training intensities involved in different medication-assisted interventions and training contexts, make the results potentially biased in addition to the asymmetrical gender ratio of some incorporated studies. Whereas the study by A. Kautzky-Willer et al. [1] also demonstrated some variations in IL-6 and CRP levels between genders, as well as inconclusive changes in the inflammatory response levels during post-intervention training [17].

In addition, a significant portion of the studies did not specify the dietary structure of the subjects during the intervention, and the subjects remained on regular metformin during training, which may be a source of the high degree of heterogeneity between studies. AT and CT were superior to RT and HIIT in reducing IL-10 and IL-6 levels in MEPT2D, similar to previous studies [15,30], possibly because local muscle fiber damage induced by RT persists 12–72 h after exercise [33], whereas in the RT studies selected for this meta-analysis, 24–48 h after exercise was the time when most of the blood was collected in the studies, when local sterile inflammation caused by muscle fiber microscopic damage leaves a range of inflammatory factors (e.g., IL-6, IL-10) still in a state of dynamic fluctuation. As for TNF-α, we found that HIIT was able to reduce TNF-α levels to some extent in MEPT2D, which may be related to the local sterile inflammation triggered by the accumulation of metabolic wastes due to the high-intensity operation of the anaerobic energy supply system, as found in the study by Edite Teixeira de Lemos et al. [6]. In contrast to the results that high IL-6 and IL-10 levels in abnormal states of the body promoted the inflammatory response, the elevated IL-10 and IL-6 levels that occurred after training instead suppressed TNF-α levels by modulating the sensitivity of anti-inflammatory factor, thereby ameliorating the chronic inflammatory response.

Moreover, different training frequencies and training durations had different effects on inflammatory factor levels in MEPT2D. M.L. Jorge [17] and G. Annibalini [24] found in their study that prolonged (60–90 min) AT, RT, and CT training had a rather limited effect on improving inflammatory factors in MEPT2D, which was related to excessive exercise capacity [34]. The progressive and pathological decline of the energy metabolic system is common in MEPT2D [35], and prolonged systematic training tends to result in a large accumulation of metabolic waste products such as lactate, which further affects short-term metabolic efficiency and internal environmental homeostasis [36], and even causes aseptic acute inflammation at the cellular level, which affects the levels of pro-inflammatory factors in the subjects [37]. In addition, prolonged moderate intensity training also results in an excessive loss of electrolytes, water, and sugar, which may lead to an acid-base imbalance and blood concentration in the subjects [38], and excessive glycogen depletion may have a negative impact on the insulin status of MEPT2D [39]. Therefore, exercise interventions lasting more than 90 min in a single session are not recommended for middle-aged and elderly patients with type 2 diabetes.

JP [31] showed that prolonged HIIT exercise (60–90 min) was more effective in improving pro-inflammatory factors than conventional training sessions (40–60 min), which may be related to the exercise pattern of HIIT, a complex high-intensity, short-interval aerobic exercise that involves a variety of coordinated whole-body muscle groups. Compared with traditional AT, HIIT involves multiple cycles of movement and body positions during prolonged exercise, which mobilizes muscle glycogen from a wide range of muscle groups for energy, while avoiding over-stimulation of a single muscle group that can lead to local lactic acid build-up and affect the training effect. This not only improves glycolipid levels, but also optimizes blood glucose profiles by modulating energy system sensitivity and insulin sensitivity, thereby improving pro-inflammatory factors in MEPT2D [40]. In addition, higher training frequency (3–5 sessions/week) had a more significant effect on inflammatory factor levels in MEPT2D, as confirmed by P. Lucotti [18] and N.P. Kadoglou [20], who showed that increasing the frequency of training within a cycle seems to be better than increasing the length of a single training session. Improving the level of inflammatory factors in subjects, which was related to the regulation of energy metabolic systems associated with exercise. While longer training sessions may lead to a range of negative symptoms and hinder the improvement of anti-inflammatory factor levels [41], higher training frequencies can increase circulatory capacity while ensuring adequate replenishment of the energy system and recovery of myocyte contractility and cardiorespiratory capacity. On the one hand, such a training schedule ensures the quality of a single exercise intervention; on the other hand, it increases the total intensity of training to the extent that the subject’s body can withstand it, thus improving inflammatory factor levels in MEPT2D.

In addition, it was found that the effect of exercise on the improvement of inflammatory factors in MEPT2D was also influenced by the changes in BMI during the study period. Not only were energy metabolism and the insulin system regulated by exercise during the exercise intervention, but also BMI and the percentage of adipose tissue decreased in most subjects. Adipose tissue has an effect on inflammatory factor levels [42], and numerous studies have shown that obese individuals have higher levels of inflammatory factors than the general population and are therefore more likely to have diabetes, cardiovascular disease, metabolic disorders, and kidney disease [43]. Therefore, the reduction in BMI from the intervention may contribute to the effect of exercise on the levels of inflammatory factors in MEPT2D.

Different training intervention cycles may also be at the root of the insignificant training effects in individual studies. The period of intervention for the included studies in this meta-analysis varied from three weeks to one year, while some studies have shown that a training interval of at least 12 weeks is required to achieve a significant decrease in TNF-α levels [7]. Lastly, for CRP, we found that CT was more advantageous than alternative training options (AT, RT, and HIIT). Unlike TNF-α, IL-6, and IL-10, CRP is a plasma protein in the acute inflammatory phase and is synthesized by adipocytes in addition to the liver. CT not only reduces CRP levels through lipid metabolism, but also enhances the metabolism of skeletal muscle via the triggering of AMPK (5’ adenosine monophosphate-activated protein kinase) signaling pathway [32]. Furthermore, the sensitivity and capacity of the backup energy supply system is enhanced by the supplemental energy supply pattern, which contributes to the modulation of both hepatic glycogen synthesis and fat catabolism, slowing muscular wastage and enhancing all-day body metabolism, thereby improving inflammation levels.

## 5. Current Research Limitations

This study adhered to the prescribed procedures of PRISMA, but there was some heterogeneity in the results since relatively few HIIT studies were enrolled. Moreover, MEPT2D tend to already be in persistent inflammatory physical states due to the relatively long duration of the disease (mean age >3 years), high blood glucose levels, and long-term lack of exercise interventions. In a number of studies, subjects were synchronized with adjunctive pharmacological interventions, and these factors in turn may have disrupted the ability to evaluate variations in inflammatory factors. In addition, the sensitivity of the anti-inflammatory system varies by age of disease, resulting in different baseline results for the same component of the training intervention. Exercise interference involves different types of training motions and phase differences in intensity, since the study may be biased by other unseen factors due to different practice settings.

## 6. Conclusions

AT, RT, CT, and HIIT all reduced the levels of inflammatory response factors of CRP, TNF-α, IL-6, and IL-10 in MEPT2D, and CT was outperformed by alternative training modalities in lowering the factors of inflammation response of CRP, IL-6, and IL-10 in MEPT2D. In contrast, HIIT was superior to other exercise modalities in decreasing the inflammatory response factor of TNF-α in MEPT2D.

According to the findings, training modalities were found to be beneficial in lowering the levels of inflammatory factors in MEPT2D, which confirmed the usefulness and scientific validity of exercise training in relieving chronic low-grade inflammation in MEPT2D. Combined with the results of the subgroup analysis, CT is the optimal training modality to improve the inflammatory response in MEPT2D, and CT can be an entry point for future researchers to explore the most optimal training modality, periodicity, frequency, and intensity with appropriate modality interventions and standardized pharmacological interventions according to the patients’ age.

## Figures and Tables

**Table 1:** General characteristics of the selected research literature.

NO.	Authors Nation	Year	Age/BMI		Sample Size (Male/Female)	Training Mode	Frequency	Duration	Other Intervention	Outcomes
			Control Group	Intervention Group	Control Intervention Group Group					
1	Balducci S et al. [15] <Italy>	2009	60.6 ± 9.3 (30.9 ± 1.1)	64.3 ± 8.1 (30.1 ± 1.0)	22 (14/8) 20 (12/18)	AT, CT	60 min/session 3 sessions/week	12 months	Diet control and medication	TNF-α, CRP, IL-6I L-10
2	Okada S et al. [16] <Japan>	2010	64.5 ± 5.9 (24.5 ± 2.9)	61.9 ± 8.6 (25.7 ± 3.2)	17 (11/6) 21 (10/11)	RT	60 min/session 3 sessions/week	12 weeks	Diet control and medication	CRP
3	Jorge ML et al. [17] <Brazil>	2011	53.4 ± 9.8 (29.59 ± 4.9)	52.1 ± 8.7 (30.42 ± 3.53)	12 (4/8) 36 (14/22)	AT, RT, CT	90 min/session 3 sessions/week	12 months	None	TNF-α, CRP, IL-6
4	Lucotti P et al. [18] <Italy>	2011	58.1 ± 9.9 (38.8 ± 4.5)	61.5 ± 11.5 (37.5 ± 4.2)	27 (10/17) 20 (7/13)	AT, CT	60 min/session 5 sessions/week	3 weeks	diet control	TNF-α
5	Abd El-Kader SM et al. [19] <Saudi Arabia>	2013	44.1 ± 5.9 (33.2 ± 2.8)	43.6 ± 6.2 (34.5 ± 1.5)	40 (17/23) 40 (19/21)	AT	60 min/session 3 sessions/week	12 weeks	None	TNF-α, IL-6
6	Kadoglou NP et al. [20] <Greece>	2013	57.9 ± 7.2 (32.1 ± 2.95)	57.4 ± 5.7 (32.24 ± 3.16)	AT:21 (6/15) 24 (7/17) RT:23 (7/16) CT:22 (5/17)	AT, RT, CT	60 min/session 4 sessions/week	24 weeks	Metformin etc.	CRP
7	Kim KB et al. [21] <Korea>	2014	52.5 ± 1.8 (26.41 ± 2.42)	51 ± 1.38 (25.92 ± 2.97)	8 (0/8) AT:8 (0/8) CT:8 (0/8)	AT, CT	60 min/session 3 sessions/week	12 weeks	None	CRP, IL-6
8	Mavros Y et al. [22] <Australia>	2014	69.2 ± 6.3 (31.1 ± 4.8)	67.2 ± 4.9 (31.1 ± 4.8)	47 (24/23) 41 (22/19)	RT	60 min/session 3 sessions/week	12 months	Diet control and medication	CRP
9	Abd El-Kader SM et al. [23] <Saudi Arabia>	2015	43.6± 6.2 (31.65 ± 5.53)	44.1 ± 5.9 (30.87 ± 5.76)	25 (un) 25 (un)	AT	60 min/session 3 sessions/week	12 weeks	None	TNF-α, IL-6
10	Annibalini G et al. [24] <Italy>	2017	60 ± 6.8 (29.0 ± 3.8)	57 ± 9.1 (28.3 ± 1.5)	8 (8/0) 8 (8/0)	CT	90 min/session 3 sessions/week	16 weeks	Glucose-lowering drugs	TNF-α, CRP, IL-6
11	Mallarda AR et al. [25] < Australia >	2017	54.9 ± 5.3 (29.6 ± 3.6)	58.6 ± 5.0 (30.2 ± 2.7)	18 (12/6) 20 (12/8)	HIIT	70 min/session 3 sessions/week	12 weeks	None	TNF-α, IL-6, IL-10
12	Miller EG et al. [26] <Australia>	2017	66.9 ± 5.3 (32.5 ± 3.8)	66.9 ± 5.3 (31.5 ± 10.9)	18 (8/10) 18 (11/7)	RT	60 min/session 3 sessions/week	12 months	None	IL-10
13	Martins FM et al. [27] <Brazil>	2018	65 ± 6.3 (29.2 ± 7.1)	64.3 ± 6.7 (27.0 ± 6.2)	8 (0/8) 8 (0/8)	HIIT, CT	60 min/session 3 sessions/week	12 weeks	None	CRP, IL-6
14	Dadrass A et al. [28] <Iran>	2019	53.2 ± 8.1 (27.7 ± 3.55)	53.75 ± 8 (27.94 ± 4.43)	12 (un) 12 (un)	RT	60 min/session 3 sessions/week	12 weeks	VitaminD	TNFα, CRP, IL-6
15	Rech A et al. [29] <Brazil>	2019	68 ± 6.5 (28.3 ± 3.1)	70.5 ± 7.4 (28.46 ± 3.2)	21 (10/11) 17 (10/7)	AT, RT	60 min/session 3 sessions/week	12 weeks	None	TNF-α, CRP, IL-6I L-10
16	Abd El-Kader SM et al. [30] <Saudi Arabia>	2020	42.2 ± 4.9 (30.58 ± 2.76)	41.5 ± 5.2 (31.16 ± 2.89)	50 (32/18) 50 (34/16)	AT	60 min/session 3 sessions/week	12 weeks	Diet control	TNF-α, IL-6
17	Magalhães, J.P. et al. [31] <Portugal>	2020	59 ± 8.1 (30.7 ± 5.0)	58.9 ± 7.5 (31.1 ± 5.0)	27 (14/13) 13 (9/4)	HIIT	90 min/session 3 sessions/week	12 months	Dietary counselling	TNF-α, CRP, IL-6
18	Su X et al. [32] <China>	2022	63.6 ± 2.6 (25.67 ± 0.96)	64 ± 1.9 (24.9 ± 0.67)	13 (0/13) 14 (0/14)	CT	60 min/session 3 sessions/week	12 weeks	hypoglycemic drugs	TNF-α, CRP, IL-6

Un—unknown; AT—Aerobic training; RT—Resistance training; CT—Combined training; HIIT—High-intensity interval training; CRP—C-reactive protein; TNF-α—Tumor necrosis factor alpha; IL-6—interleukin 6; IL-10—interleukin 10.

## Data Availability

The experimental data supporting this study are publicly available.
